# Risk assessment on-a-chip: a cell-based microfluidic device for immunotoxicity screening†

**DOI:** 10.1039/d0na00857e

**Published:** 2020-12-18

**Authors:** Arianna Oddo, Mariana Morozesk, Enzo Lombi, Tobias Benedikt Schmidt, Ziqiu Tong, Nicolas Hans Voelcker

**Affiliations:** Drug Delivery, Disposition and Dynamics, Monash Institute of Pharmaceutical Sciences, Monash University Parkville Victoria 3052 Australia nicolas.voelcker@monash.edu tommy.tong@monash.edu; Universidade Federal de São Carlos, Departamento de Ciências Fisiológicas Rod. Washington Luiz, Km 235, São Carlos 13565-905 São Paulo Brazil; Future Industries Institute and UniSA STEM, University of South Australia Mawson Lakes 5095 South Australia Australia; Department of Applied Chemistry, Reutlingen University Alteburgstraße 150 72762 Reutlingen Germany; Commonwealth Scientific and Industrial Research Organisation (CSIRO) Clayton Victoria 3168 Australia; Melbourne Centre for Nanofabrication, Victorian Node of the Australian National Fabrication Facility Clayton Victoria 3168 Australia; Department of Materials Science & Engineering, Monash University Clayton Victoria 3168 Australia

## Abstract

Nanomaterials are widely used in industrial and clinical settings due to their unique physical and chemical properties. However, public health and environmental concerns have emerged owing to their undesired toxicity and ability to trigger immune responses. This paper presents the development of a microfluidic-based cell biochip device that enables the administration of nanoparticles under laminar flow to cells of the immune system to assess their cytotoxicity. The exposure of human B lymphocytes to 10 nm silver nanoparticles under fluid flow led to a 3-fold increase in toxicity compared to static conditions, possibly indicating enhanced cell–nanoparticle interactions. To investigate whether the administration under flow was the main contributing factor, we compared and validated the cytotoxicity of the same nanoparticles in different platforms, including the conventional well plate format and in-house fabricated microfluidic devices under both static and dynamic flow conditions. Our results suggest that commonly employed static platforms might not be well-suited to perform toxicological screening of nanomaterials and may lead to an underestimation of cytotoxic responses. The simplicity of the developed flow system makes this setup a valuable tool to preliminary screen nanomaterials.

## Abstract 2

This study presents the development of a microfluidic device to assess the cytotoxicity of nanoparticles on human immune cells.

## Graphical abstract

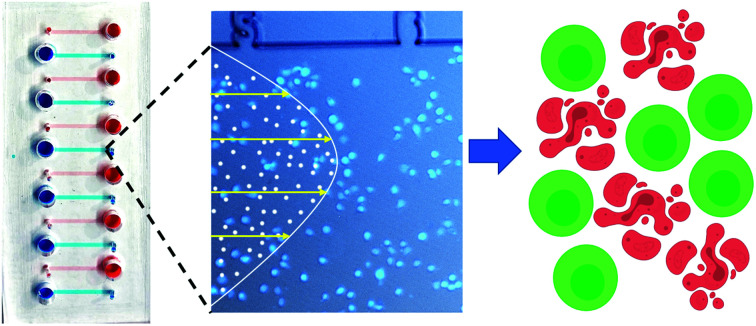

## Introduction

Nanoparticles (NPs) are used to improve the quality and performance of a wide range of consumer and industrial products. Owing to their tunable physical and chemical properties (*e.g.* surface chemistry and electrical conductivity), products containing NPs display unique characteristics, such as enhanced thermal stability, efficiency, and robustness.^1^ For example, titanium dioxide NPs and zinc oxide NPs are added to sunscreens to reflect and scatter UV radiations, while gold NPs have been successfully employed for biomedical applications.^2^ Silver nanoparticles (AgNPs) are among the most commercialized nanomaterials due to their enhanced antibacterial property.^3^ Despite the attractive properties that engineered nanomaterials bring forth, growing evidences also suggest that some NPs may interact with the immune system, leading to the production of inflammatory cytokines and chemokines, and ultimately inducing immunotoxic effects.^4,5^ Some inorganic NPs were also found to induce cytotoxic effects in peripheral blood lymphocytes, which include T cells, B cells, and natural killer cells.^6,7^ Since human exposure to nanomaterials is on an increasing trajectory^8^ and the process of particle distribution and elimination *in vivo* starts as soon as NPs reach the blood flow,^9–11^ the consequences of NPs interacting with the immune system need to be determined to evaluate their biocompatibility.^12^ In the regulatory context, the final evaluation on hemocompatibility and immunoreactivity testing relies on *in vivo* studies.^13,14^ However, given the intrinsic limitations and inconclusive outcome of animal models, over the past few decades there has been an increased scientific interest in the development of non-animal model alternatives to mimic realistic exposure scenarios.^15^ To date, most of the preliminary immunotoxicity testing carried out *in vitro* fails to mimic blood flow and neglects the shear stress to which blood cells are exposed. In the human body, shear stress ranges between 0.1 and 3 Pa in blood vessels, depending on their size, and from 0.06 Pa to 1.2 Pa in lymphatic vessels.^16^ Thus, the development of fit-for-purpose methods to rapidly assess the hemocompatibility and immunotoxicity of NPs is a high priority among public health authorities and the scientific community.^17^

Advances in tissue engineering, microfluidic technologies, and microfabrication have recently led to the development of novel *in vitro* platforms, including complex organ-on-a-chip models and simpler cell biochip devices (CBDs).^18^ Microfluidic models allow for continuous fluid perfusion inside microchannels plated with different cell types, are amenable to higher throughput, and capable of high resolution microscopy and real-time single-cell tracking.^19,20^ Microfluidic devices have also been used to study cell–NP interactions, as NPs can be delivered under a laminar flow profile in the microchannels. Consequently, these models can provide valuable information on NP internalization, dosimetry, and cytotoxicity.^21^ Compared to conventional 96-well plate platforms, fluid flow maintains a homogeneous distribution of NPs, reducing phenomena such as NP aggregation and gravitational settling, and thus leading to a more precise NP dosimetry.^22^ As an early stage screening platform, microfluidic devices can support researchers and regulatory authorities in prioritizing the need for further toxicity testing.^23,24^ However, in order to fulfil the potential of this emerging technology, some validation issues need to be addressed and results obtained cross-compared with traditional techniques.^25^

The aim of this study was to develop and validate a proof-of-concept CBD to assess the cytotoxic effect of NPs administered under fluid regime on cells of the immune system, allowing to recapitulate more closely the conditions occurring *in vivo* such as blood flow. Therefore, we developed a system and methodology to investigate whether the administration of commercially available AgNPs under dynamic flow regime would induce differences in cell cytotoxic responses, when compared to conventional assays. To take full advantage of the miniaturization of CBDs and obtain more precise results, we developed a system capable of achieving single-cell tracking and monitoring cell population variations throughout the experiments.

## Materials and methods

### AgNPs quality controls

Polyvinylpyrrolidone (PVP)-coated AgNPs with a diameter of 10 nm were purchased from NanoComposix Inc., USA. Routine NP quality controls were carried out according to the manufacturer's recommendations. Several characterization techniques were employed to assess the stability of AgNPs, including UV-Vis spectrophotometry, inductively coupled plasma mass spectrometry (ICP-MS), transmission electron microscopy (TEM), and cryo-transmission electron microscopy (Cryo-TEM). Experimental details for each analytical technique are reported in ESI† following the protocols from the manufacturers (ESI Methods).

### Cell exposure to AgNPs in a conventional assay

The toxicity of AgNPs to B cells was first assessed using a conventional approach, namely a 96-well plate and a commercially available viability kit. An acute human B cell leukemia cell line (PJ3.HR1K, ATCC) was cultured in complete media: RPMI 1640 (Sigma-Aldrich) supplemented with 10% fetal bovine serum (Thermo Fisher), 100 units per mL penicillin and 100 g mL^−1^ streptomycin (Invitrogen), 4.5 g L^−1^d-glucose, 1x Glutamax (Thermo Fisher) and 1 mM sodium–pyruvate (Thermo Fisher). The cell line was maintained at 37 °C and 5% CO_2_ in a humidified cell culture incubator. An ATP-based luminescent cell viability assay, CellTiter-Glo® (Promega, USA) was used to determine cell viability according to the manufacturer's protocol. Briefly, a calibration curve was established by measuring the fluorescence intensity *vs.* the nominal cell number of HR1K seeded, and was subsequently used to determine the percentage of viable cells after exposure to AgNPs. To assess the cytotoxicity of AgNPs, 10^4^ cells were seeded in each well. A 100 μg mL^−1^AgNP stock solution was prepared and an appropriate volume added in each well to achieve the following concentrations: 1, 5, 10, 12.5, 15, 20, 25, 30, 40, and 50 μg mL^−1^. Complete RPMI medium was added to each well to reach a final volume of 60 μL. After 5 h, 60 μL of the CellTiter-Glo® solution was added to each well. The 96-well plate was placed on an orbital shaker for 2 min, and further incubated for 10 min at RT. Luminescence intensity was measured on an EnSpire Multimodal Plate Reader (PerkinElmer). Viability was normalized against the untreated control.

### Fabrication and assembly of microfluidic chips and of PDMS wells

The silicon master for the CBD was designed and fabricated using a conventional photolithography procedure according to a protocol previously developed by our group and reported in ESI†.^26^ Microfluidic chips were fabricated using polydimethylsiloxane (PDMS, Sylgard™ 184 Silicone Elastomer Kit, DOW) mixture (10 : 1, w/w base to catalyst ratio). The mixture was poured onto the silanised silicon master and degassed under vacuum, then cured at 80 °C for 24 h. A surgical knife was used to cut out the PDMS chip, which was then peeled from the silicon wafer. Finally, 1 mm and 3 mm diameter Harris Uni-Core punctures (Ted Pella, USA) were used to create ports at the ends of the channels.

Since the HR1K cell line expresses the CD20 antigen, glass cover slides can be functionalized with the anti-CD20 antibody to capture cells.^27^ Thin glass cover slides (#1, 24 mm × 60 mm, Menzel-Gläser) or standard microscope glass slides (75 mm × 25 mm × 1 mm) were treated with 10% v/v 3-(glycidoxypropyl)-trimethoxysilane (GPTMS, Sigma) in anhydrous toluene to enhance protein adsorption.^28^ To assemble the cell culture devices, the silanized glass slide was either bonded to a PDMS chip by oxygen plasma treatment for irreversible bonding (Harrick Plasma) or by sandwich bonding for a reversible structure. In the sandwich bonding procedure, standard microscope slides were used, as they were thick enough to prevent breakage at dissemble process.^29^ When plasma bonding was used, the central part of the cover slide was temporarily covered with a PDMS mask to protect the silanized surface.^27^ The mounted device was sterilized under UV light for 20 min before loading the microchannels with anti-CD20 antibody (0.1 mg mL^−1^, MabThera®, Roche), and stored in a humidified Petri dish at 4 °C overnight.

**Fig. 1:**
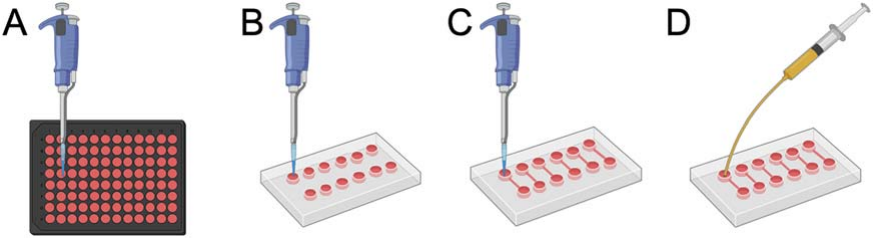
Four platforms were used to assess toxic effects of AgNPs on HR1K cells: (A) a routinely used polystyrene 96-well plate, (B) PDMS wells on glass slide, (C) microfluidic devices under static conditions (CBD_static_), and (D) microfluidic devices under laminar flow connected to a syringe pump (CBD_flow_).

A hybrid platform constructed with PDMS resembling the structure of a 96-well plate (Fig. 1A) was fabricated and referred to as “PDMS wells” (Fig. 1B). In short, a mixture of PDMS was prepared and poured onto a Petri dish, degassed under vacuum, and cured as reported above. A rectangular piece of cured PDMS (∼20 mm × 50 mm) was carved out and an array of wells were created using a 3 mm puncture. Whilst 96-well plates and PDMS wells were used for the administration of AgNPs under static conditions, microfluidic chips were used for both static (CBD_static_) and flow (CBD_flow_) conditions (Fig. 1C and D, respectively).

### Cell exposure to AgNPs in non-conventional setups, data analysis, and generation of dose–response curves

Several platforms were constructed to study the effect of every modification introduced in the system, such as the immobilization of B cells (Table 1).

**Table 1:** Summary of the different platforms included in this study and parameters assessed

Setup	Nomenclature	Parameter investigated
96-well plate	96-well plate	Toxic effect of AgNPs in a conventional setup, with HR1K cells in suspension and static conditions
PDMS wells	PDMS wells	Effect of the immobilization of HR1K cells and toxic response to AgNP exposure
Microfluidic device (static)	CBD_static_	Toxicity of AgNPs to immobilised HR1K cells seeded in microchannels
Microfluidic device (static, with post-exposure time)	CBD_static,P_	Delayed onset of toxic effect following a 19 h post-exposure time in cell culture medium
Microfluidic device (flow)	CBD_flow_	Toxicity of AgNPs administered under laminar flow to immobilised HR1K seeded cells in microchannels

HR1K cells were pre-stained with a 1 μM CellTracker™ Blue (CTB, Thermo Fisher) solution to be better suited for automated counting.^30^ Cell seeding density was adjusted to 8 × 10^6^ cells per mL for CBDs or to 5 × 10^5^ cells per mL for PDMS wells. 10 μL of cell suspension was added to each PDMS well, while 20 μL of cell suspension was pipetted into the 3 mm port of CBD_static_ or CBD_flow_ and 10 μL suspension removed from the 1 mm port to draw the cell suspension through the channel.

To ensure that cells seeded in the CBDs or in PDMS wells were properly immobilized to the glass surface, the devices were incubated for 1 h at 37 °C and 5% CO_2_ after cell seeding. AgNP solution were prepared in complete RPMI medium to achieve the following concentrations: 0.1, 0.5, 1, 5, 10, 15, 20, 25, 30, 35, 40, 60, 80 and 100 μg mL^−1^. For CBDs under flow condition, particle concentrations up to 25 μg mL^−1^ were tested on chip as 25 μg mL^−1^ of AgNP concentration already induced substantial cell death and no higher AgNP concentrations were tested.

For experiments performed in CBD_static_, 30 μL of NP suspension was manually pipetted into the 3 mm port of the chip, and then 10 μL were slowly removed from the 1 mm port. This process was repeated once more. For experiments performed in PDMS wells, 60 μL of AgNP solution was added to each well. Fluorescence and bright field images were acquired at this stage to quantify the number of cells seeded (cells stained with CTB, *t* = 0 h). The position along the microchannel was also recorded to monitor changes in the cell population throughout the experiment. A stage top incubator on an EVOS FL Auto Imager microscope (Thermo Fisher) equipped with a live cell unit (humidity, temperature, and CO_2_ controls) was used and images were acquired at 10× magnification. After image acquisition, CBDs and PDMS wells were transferred to an incubator (5% CO_2_ and 37 °C) for the incubation of 5 h. For experiments performed in CBD_flow_ platforms, a NE-1200 Syringe Pump (New Era Pump Systems Inc, USA) was employed. Briefly, 1 mL syringes (Injekt®-F, B|Braun, Germany) were attached to a needle (Terumo Needle, Belgium), which was then inserted into a Tygon tubing (Cole-Parmer, USA) and connected to the 1 mm port of the CBD. The fluid flow rate was set to 0.2 μL min^−1^ for a total administration volume of 60 μL. After 5 h, the NP suspension was removed and replaced with a propidium iodide (PI, 20 μg mL^−1^, Themo Fisher) and Hoechst 33342 (1 μg mL^−1^, Thermo Fisher) staining solution. After a 5 min incubation at RT, bright field and fluorescence microscopy images were acquired. The Fiji software (version 2.0.0-rc-65/1.51w) was used to automate batch processing and to calculate the number of cells stained with either CTB, PI, or Hoechst.^31^ Fiji scripts, reported in ESI,† were written to extrapolate cell viability statistics from the acquired images. The measure of cell integrity was used to assess cell viability. The 50% cytotoxic concentration (CC_50_) values were calculated using a non-linear regression analysis (‘[inhibitor] *vs.* response–variable slope’) from GraphPad Prism (GraphPad Software, Inc., USA). CC_50_ was defined as the NP concentration causing 50% cell death (expressed as the sum of cells stained by PI and detaching from the glass substrate).^32^ Viability was normalized to the untreated control.

Finally, another set of experiment was designed to evaluate the delayed toxicity onset of AgNPs. To this end, after the 5 h exposure under static conditions in CBDs, AgNPs were replaced with complete RPMI medium and the microfluidic devices were moved to an incubator at 37 °C and 5% CO_2_ for 19 h (CBD_static,P_). Cell staining and viability assessment were carried out as described above. These two different sets of experiments allowed us to monitor immediate toxic response (*t* = 5 h) and also the effect of NP internalization after a longer period of incubation (*t* = 19 h), as summarized in Table 1.

### Darkfield hyperspectral microscopy

A reversible bonding of the PDMS chip to a silanised standard microscope glass slide was employed to prepare samples suitable for darkfield microscopy. After exposure to AgNPs both under static and flow conditions, cells were washed with PBS and fixed with 4% paraformaldehyde (Sigma) for 10 min. The microchannels were then washed with PBS and the PDMS chip was carefully detached from the glass slide. A drop of glycerol was applied before placing the microscope glass slide recovered from the chip onto a new cover slide. Images were acquired on a CytoViva Hyperspectral Imaging System (CytoViva Inc.), consisting of an Olympus BX53 darkfield microscope equipped with a high-resolution CytoViva 150 adapter, a motorized stage, a visible-near infrared hyperspectral camera system and a 150 W halogen light source. Images were captured under darkfield illumination using a 60× 1.2 NA oil objective, while data obtained and analyzed using ENVI 4.4 software.

### Statistical analysis

Experiments were conducted in triplicate (*n* = 3) unless otherwise stated. All statistical tests were conducted using Prism7 software (Graph Pad, Inc.). Error bars presented in charts equal ±1 standard deviation (SD). Statistical differences were tested by non-parametric Kruskal–Wallis 1-way ANOVA test or by Welch's *t* test. Significance was determined by a *p* value less than 0.05.

## Results

### Stability of AgNPs

To establish and validate the CBD approach, well-characterized, commercially available AgNPs were used as a model toxic nanoparticles.^33,34^ PVP-coated AgNPs are known to induce higher cell uptake and cytotoxicity compared to bare AgNPs because of the enhanced stability and dispersibility in biological media provided by the polymer.^35^ Consequently, decreased agglomeration is associated with small, monodispersed particles and results in higher toxicity.^36^ Several studies have shown that nanosilver toxicity is affected by the availability of Ag^+^ ions in culture medium.^37,38^ Thus, to exclude that the toxic effects observed were a consequence of extracellular Ag^+^, we performed several characterization steps.^39^ The size of AgNPs in MilliQ was routinely validated by UV-Vis spectroscopy, as recommended on the product description and the peak wavelength compared to the reference values (380–405 nm). ICP-MS measurements showed that silver was mainly present in the form of colloidal silver (95.5%) over its ionic form after a 5 h incubation in complete RPMI medium (ESI Table S1†). TEM images of the stock AgNPs in ultrapure H_2_O also showed monodispersed particles, with a nominal size of 9.9 ± 2.3 nm (ESI Fig. S2A†), which correlated well with the specifications provided by the manufacturer. The UV-Vis spectra of AgNPs in complete RPMI showed a red shift of the LSPR after a static incubation period of 5 h (ESI Fig. S2D and E†), as indicated by the increase of an absorbance peak at 550 nm over time. This can be interpreted as the tendency of AgNPs to partially agglomerate under static conditions.

In order to determine whether the incubation of AgNPs under fluid regime would alter the size distribution, we further characterized AgNPs under static and flow conditions. Cryo-TEM images of AgNPs dispersed in PBS (ESI Fig. S2B, C and F†) showed particle monodispersity. The corresponding particle size distribution (9.2 ± 2.6 nm for NPs incubated under flow and 10.1 ± 2.9 nm in a static platform), calculated by measuring the size of 150 individual NPs, were within the specifications provided by the manufacturer. Thus, these results showed that PVP-AgNPs are stability in highly saline solutions and represent a suitable model to compare their cytotoxicity between different platforms.

### CBD design and setup

**Fig. 2:**
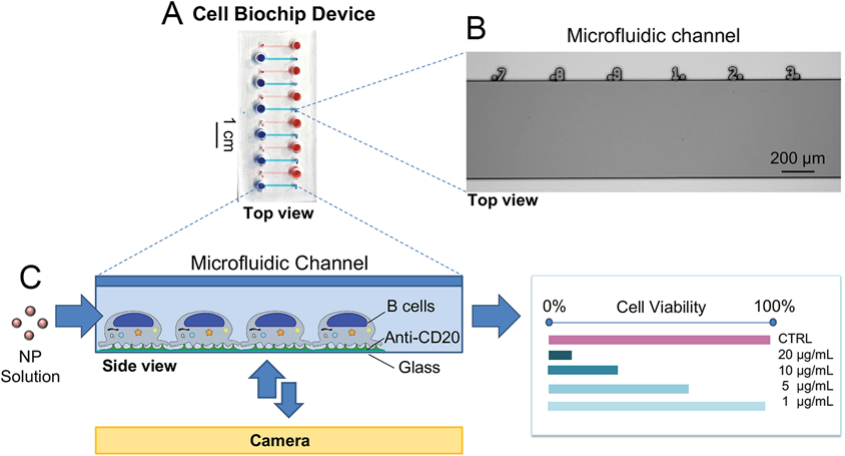
The proof-of-concept microfluidic CBD to assess the cytotoxicity of NPs on human immune cells. (A) The 12 microchannels are visualized with food color dye. (B) Top view of a portion of a microchannel from the silicon master used to prepare the PDMS chips *via* soft lithography, with numbers engraved serving as a microruler. (C) Schematic representation of the CBD used to evaluate the cytotoxicity of nanoparticles. (B) Cells are immobilized inside the microchannels and exposed to AgNP solutions; cell viability is assessed *via* a fluorescence-based assay.

A proof-of-concept microfluidic device and a semi-automated methodology for viability quantification were developed to assess cytotoxic effects of AgNPs administered either under static conditions or under fluid flow. The microfluidic device was designed with 12 parallel microchannels (600 μm wide, 70 μm high and 1.3 cm long). Food coloring dye was used for visualization of the microchannels (Fig. 2A). In order to track cells with higher accuracy, we incorporated engraved numbers along the top edge of the microchannels serving as fiduciary markers to identify the same position in a microchannel at different time points and achieving single-cell tracking (Fig. 2B). The viability of HR1K cells in the CBD was evaluated using a fluorescence-based assay following the exposure to NPs (Fig. 2C) and the corresponding viability value was determined.

### Implementation of a semi-automated procedure to quantify cell viability

**Fig. 3:**
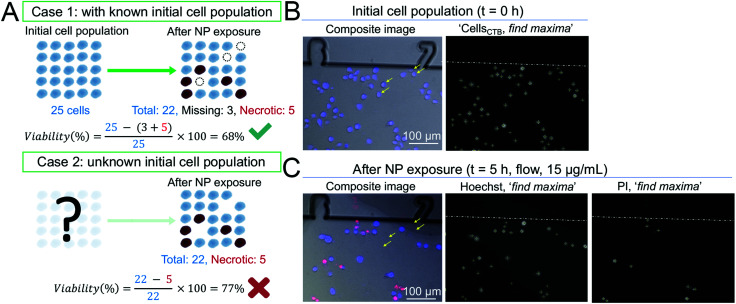
Methodology to assess cytotoxic effect of AgNPs *via* a semi-automated data analysis. (A) Examples of two hypothetical scenarios (case 1 and case 2) demonstrating the importance of tracking the initial cell population and leading to two different viability calculations. In case 1, all three parameters are known (initial cell population number = 25, necrotic cell number = 5, and missing cell number = 3). Hence, the viability after NP exposure takes into account both necrotic and missing cells and is calculated to be 68%. The viability obtained is accurate and correct (green tick). However, in case 2, the initial population is unknown. Hence, viability is calculated based on the total number of cells found at the end of the experiment (total cell number = 22) and necrotic cells (necrotic cell number = 5), as the number of missing cells cannot be determined. Consequently, in case 2, viability is equal to 77%, leading to an overestimation of 9% (red cross). (B) Composite images of B cells pre-stained with CTB (blue) at *t* = 0 h in the CBD. Cells were counted with the ‘find maxima’ Fiji function and appear with a (+) symbol in the ‘find maxima’ images. (C) Upon PI staining, necrotic cells (red) can be identified and counted automatically using the ‘find maxima’ Fiji function. The yellow arrows show that some cells detached upon exposure to toxic solutions of AgNPs under fluid regime (*e.g.* 15 μg mL^−1^) and are classified as missing cells.

With a view to develop a microfluidic platform for nanotoxicity screening applications, a semi-automated data analysis pipeline was also established, which allowed to take into account cells that detached during the experiments. Since changes in growth rate and reduction of the total number of cells are phenomena linked to cytotoxicity, changes in the total number of cells throughout the experiment should be closely monitored. As demonstrated in a hypothetical scenario in Fig. 3A, disregarding the number of the initial population and of cells lost throughout the experiment could influence the calculated percentage of viable cells, and consequently the dose–response curves. To address this issue, conventional *in vitro* platforms routinely used to assess cytotoxicity rely on internal standards or on the establishment of calibration curves. However, they require a large number of cells and do not allow to track in real-time the changes in overall cell population. In contrast, microfluidic devices allow to achieve single-cell tracking and can closely monitor small variations in the initial cell populations due to the fiducials engraved along the microchannels.

Toxicity data obtained from microfluidic devices, hence, can benefit from improved accuracy. To this end, four parameters were acquired to determine the CC_50_ values: initial cell population (Cells_CTB_), total cell population at the end of the experiment (Cells_Hoechst_), cell population that detached due to compromise membrane (Cells_miss_), and necrotic cells population counted at the end of the experiment (Cells_PI_). Cell viability was calculated according to eqn (1):1
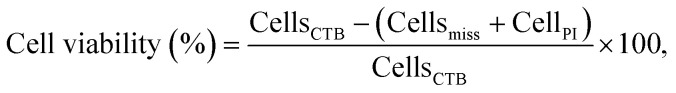
where Cells_miss_ = Cells_CTB_ − Cells_Hoechst_ cell viability was calculated as the percentage of viable cells over the initial population of cells in the same region of a microchannel. The number of viable cells was calculated based on the initial cell population (Cells_CTB_) and the sum of cells that either detached from the chip (Cells_miss_) or necrotic cells stained by PI (Cells_PI_). The variables from eqn (1) (Cells_CTB_, Cells_PI_, Cells_Hoechst_) were determined by processing the fluorescence microscopy images with a Fiji script. The numbers of total cells and of necrotic cells determined with the ‘find maxima’ function (Fig. 3B and C) reflected with good accuracy those that can be visualized in the respective composite image. However, under toxic conditions (*e.g.* administration of a 15 μg mL^−1^ AgNP solution under flow regime), cells with a compromised membrane detached after NP exposure as indicated by yellow arrows in Fig. 3C and ESI Fig S1A.† However, as shown in ESI Fig. 1B,† no cell detachments or compromised cell membranes were observed for the negative control (HR1K cells exposed to media only), demonstrating that shear force alone did neither affect cell viability nor caused a reduction in the overall cell population.

### Dynamic administration of AgNPs induces higher cytotoxicity in comparison to static assays

**Fig. 4:**
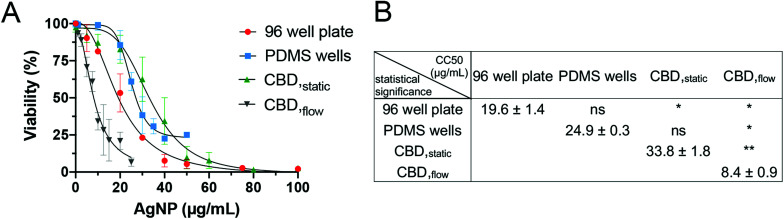
Dose–response curves for incubation of HR1K cells with AgNPs obtained in different platforms. (A) A conventional 96-well plate assay (CellTiter-Glo®), PDMS wells, CBD under static conditions (CBD_static_) and CBD under fluid flow (CBD_flow_). Error bars equal ±1 SD and *n* = 3. (B) Comparison and statistical significance analysis of the CC_50_ values.

A series of experiments was performed to validate the CBD and ensure that every modification introduced in the platform (*e.g.* the immobilization of HR1K cells) would not modify the cytotoxic effect of the NPs to the cells. Since HR1K cells are generally cultured in suspension, preliminary experiments aimed at assessing whether the anti-CD20 mediated immobilization had any effects on cell cytotoxicity. Thus, before commencing detailed studies on CBDs, commonly used viability assays were carried out in a conventional well plate format, in which HR1K cells were cultured in suspension. The aim was also to obtain a reference toxicity value in a routinely used assay, which was used to compare the data obtained under flow. We excluded routine techniques including several centrifugation steps (*e.g.* flow cytometry) as they may not be able to recover all the cells in suspension, especially in the case of damaged cell membrane and often lead to an underestimation in cytotoxicity.^28^ On the other hand, another commonly used test, the MTT assay, was also not suitable due to the long incubation time required (up to 4 h). In contrast, PI staining used in the microfluidic system only requires 5–10 min. The CellTiter-Glo® Luminescent Cell Viability Assay was chosen for the well plate platform, as it involves a short 12 min incubation time after NP exposure, similar to the time required for PI staining. The dose–response curve for AgNPs was generated using CellTiter-Glo® under static condition in a 96-well plate, and the corresponding CC_50_ value was 19.6 ± 1.4 μg mL^−1^ (Fig. 4A).

We then examined the toxicity of AgNPs in the PDMS wells format, which mimicked the conventional 96-well plate, but comprised a silanized glass substrate suitable for immobilization of anti-CD20 antibody to capture HR1K cells on the chip surface. The CC_50_ value calculated from PDMS wells was 24.9 ± 0.3 μg mL^−1^ (Fig. 4B). Compared to the CC_50_ value obtained from the 96-well plate, no significant difference was observed (Fig. 4B), which correlates with our previous studies.^28,34^ After confirming the immobilization step did not affect cell interaction with AgNPs, we performed toxicity testing in CBDs. The CC_50_ value for AgNPs administered in CBD_static_ (33.8 ± 1.8 μg mL^−1^) was also not significantly different compared to value (24.9 ± 0.3 μg mL^−1^) obtained from PDMS wells (Fig. 4B). However, the CC_50_ value obtained for CBD_static_ is significantly higher compared to the 96-well plate format. This difference could be due to the small volume of the microchannels (1.42 μL), which causes HR1K cells to be exposed overall to a smaller number of NPs, compared to the well plate format. This result highlights the importance of the number of NPs to which cells are exposed in determining the cytotoxicity. Furthermore, we were able to demonstrate that culturing immobilized HR1K cells in CBDs did not affect their viability. Finally, when AgNPs were administered in CBD_flow_ with a flow rate corresponding to a low level of shear stress of 5 × 10^−3^ Pa, we observed higher cytotoxicity in comparison to all static conditions and corresponding to the lowest CC_50_ value of 8.4 ± 0.9 μg mL^−1^ and a significant difference compared to all other cases (Fig. 4B). This result is in accordance with published studies, which demonstrated that shear stress induces an increase in cytotoxic response.^40–42^ For example, compared to traditional *in vitro* cytotoxicity assays performed under static conditions, unmodified mesoporous silica nanoparticles showed higher and shear stress-dependent toxicity to endothelial cells under flow conditions.^43^ However, the aforementioned studies have only focused on adherent cells (*e.g.* endothelial cells, lung epithelial cells, and cervical cancer cells). In contrast, whether shear stress influences the cytotoxicity of NPs on immune cells has not been reported.

**Fig. 5:**
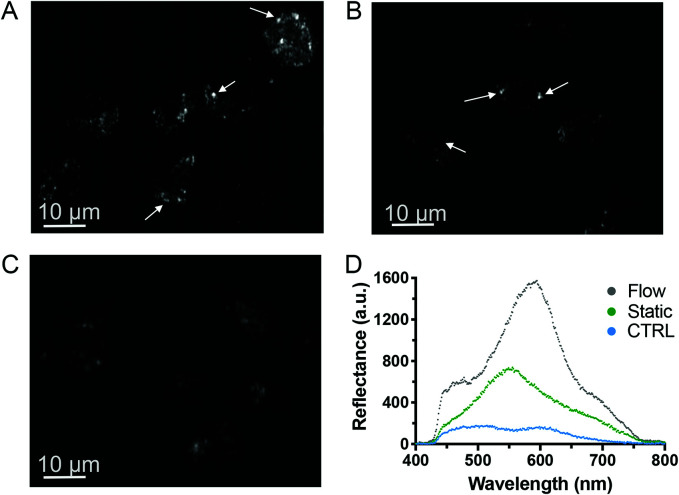
Darkfield hyperspectral microscopy of B cells exposed to AgNPs. HR1K cells were exposed to a 5 μg mL^−1^ solution of AgNPs for 5 h under flow condition at 0.2 μL min^−1^ (A) and static condition (B), or to complete RPMI media containing no AgNPs as a control (C). The white arrows indicate the high contrast between NPs (bright spots) and background cells (D) spectral comparison of three different bright areas from the three darkfield microscopy images collected.

It has recently been shown *in vivo* that NPs are internalized with greater efficacy under low shear stress (1.6 × 10^−3^ to 4 × 10^−2^ Pa).^44^ Similarly, the maximal uptake of silica NPs *in vitro* by endothelial cells has been observed at a shear stress of 0.05 Pa, which was attributed to cytoskeletal rearrangements.^45^ We have utilized a similar shear stress value of 5 × 10^−3^ Pa. The internalization of liposomes by macrophages and myoblast cells was also reported to be enhanced by shear stress, demonstrating that shear stress is critical for a realistic prediction of the uptake of particles.^46,47^ Furthermore, mechanical forces, such as shear stress, are known to trigger T cell receptors^48^ and it was recently shown that several receptors of B cells were activated by low shear stress,^49^ which could potentially induce higher NP uptake and correlate with the increased toxicity observed. However, to our knowledge, no studies to date have demonstrated whether immune cell activation influences NP uptake. Next, we investigated whether the increase in toxicity was linked to higher AgNP uptake. Given the strong light scattering features of AgNPs, we identified darkfield hyperspectral microscopy as a suitable technique to further investigate NP uptake in low scattering matrices, such as cells.^50^ Darkfield microscopy images displayed stronger scattering effect (brighter) when HR1K cells were exposed to AgNPs under fluid flow (Fig. 5A) compared to cells exposed under static conditions (Fig. 5B) or the negative control (Fig. 5C). Hence the comparison between the three images (Fig. 5D) suggests an increased NP uptake under flow conditions, which may explain the higher cytotoxicity observed.

### Post-exposure time following AgNP treatment in CBDs induced progressive cell death

**Fig. 6:**
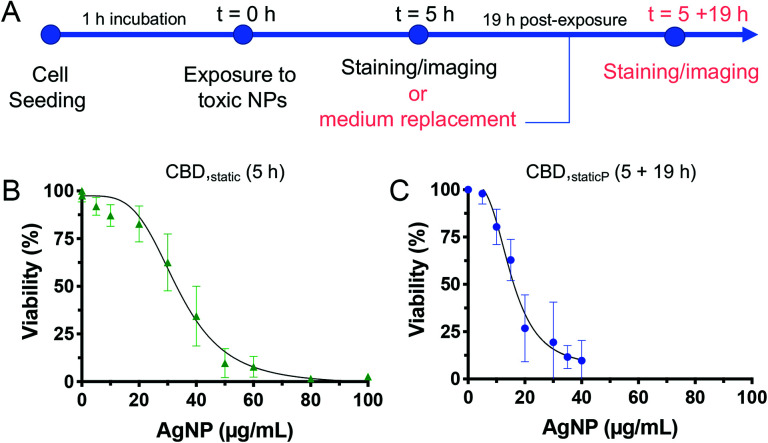
Timeframe adopted for the modified set of experiment to evaluate the effect of a 19 h post-exposure time, following the same 5 h exposure to AgNPs (A). Dose–response curve for CBD_static_ with B cells exposed to AgNPs under static conditions for 5 h (B), and CBD_static,P_ followed by 19 h post-exposure time (C). Error bars equal ±1 SD and *n* = 3.

Based on the toxicity results obtained following an acute AgNPs exposure of 5 h, we were interested to test the ability of the cells to potentially recover after the removal of AgNPs from the system. Indeed, whilst most studies assessing the cytotoxicity of chemicals focus on varying the incubation time (from a few hours up to 24 h), few of them have investigated how cell cultures react several hours after NP exposure, which may help predicting *in vivo* mechanisms such as NP clearance.^51^ Moreover, to our knowledge, the only studies investigating the ability of cells to recover following acute NP exposure were only performed in conventional cell culture platforms (*i.e.*, static incubation in well plates followed by MTT assay) and not in microfluidic devices.^52–54^ Thus, we have explored whether internalized AgNPs have a more prolonged toxic effect, or whether B cells could potentially recover from adverse effects caused by an acute exposure to toxic NPs. To this end, an additional incubation time of 19 h was included after the removal of AgNPs from the solution following 5 h exposure, and is referred to as ‘post-exposure time’ or CBD_static,P_ (Fig. 6A).^55^ Interestingly, the CC_50_ value was significantly reduced (15.2 ± 1.4 μg mL^−1^) with the added post-exposure time, in comparison to the measurements taken immediately after 5 h exposure (33.8 ± 1.8 μg mL^−1^) (Fig. 6B and C). These results highlight the importance of evaluating the ability of cells to recover, which, despite being often neglected in acute, short-term toxicity studies, is an important parameter to assess the cytotoxicity of NPs. In accordance with our results, it was found that the cytotoxic effect following administration of silica NPs on a lung papillary adenocarcinoma cell line was exacerbated after 20 h post-exposure time following a 4 h NP treatment.^40^ Finally, to assess the suitability of the microfluidic models employed throughout this study towards higher throughput, we calculated the *Z*-factor, a statistical parameter to assess the quality of high throughput screening assays. A *Z*-factor value of 0.5 and above is indicative of a robust high throughput screening assay.^56^ All *Z*-factors calculated from the 5 h assays (96-well plate, PDMS wells, CBD_static_, and CBD_flow_) have a *Z*-value >0.5, indicating their suitability for cytotoxicity screening studies (ESI Table S2†). However, the assay with the 19 h post-incubation time has a *Z*-value of 0.3 making it a marginal assay. This is due to higher standard deviations obtained for CBD_static,P_ as some cells may still be able to recover once the NP solution is replaced by fresh media.

## Discussion

Ensuring that NPs used in industrial and consumer products do not induce harmful effects is of utmost importance. Cell-based screening is generally the first step prior to lengthy and expensive *in vivo* toxicological evaluations. These initial *in vitro* assessments are very desirable, both from an economical and ethical perspectives. However, common cell-based screening is generally carried out in a well plate format, which is well-proven for testing chemicals, but is not well suited to screen NPs.^33^ Phenomena, such as gravitational settling and agglomerations, are typical characteristics of NPs in solution; which can alter NP dosimetry, leading to a poor correlation with effects observed *in vivo*. Another important aspect that conventional *in vitro* testing neglects is related to the nature of blood cells, which are constantly exposed to shear stress inside blood vessels. Some methods have been explored to maintain cells in suspension while administering AgNPs under fluid flow.^57^ For example, the Chandler Loop model, a circular conduit filled with fresh human blood sample, has been employed to study the hemocompatibility of NPs.^58,59^ However, its main limitations are the relatively large volume of NPs required and its unsuitability for screening with reasonable throughput. Another important parameter that limits the suitability of the Chandler Loop model for nanotoxicology applications is that laminar flow can only be maintained at very low rotation rates, often incompatible with size of the existing setup. Since capillaries are characterized by laminar flow due to their small length scales and high viscosity, the Chandler Loop model may induce NP aggregation when used for nanotoxicology applications.^60^

To address this technological gap, we developed a microfluidic model that can be used to study the effect of NPs on cells of the immune system and that includes shear stress. In the establishment of a proof-of-concept device, 10 nm PVP-AgNPs were employed as a model NPs, as they are well known for their cytotoxicity.^61^ Similarly, since lymphocytes are central to the development of immune responses,^62^ the HR1K cell line represented a good model to study the interactions between NPs and cells of the immune system. However, it should be noted that this approach can be easily adopted for other types of NPs and cell lines representing different routes of exposures to nanomaterials, including other blood cells in the same experiments, with single-cell tracking capabilities. Furthermore, a simple modification, such as the replacement of the fiducials engraved along the microchannels in this model with computer-recognizable micro-pattern (*e.g.* QR codes), could increase its suitability for screening a large number of NPs and cell types, as it would even allow a higher throughput image acquisition.^63^ Throughout this study, we also investigated whether B cells exposed to AgNPs for a short time could potentially recover from adverse effect. Our data together with other studies demonstrated that short, acute exposure treatments may not capture effects related to toxicity, such as altered doubling time or delayed onset of cell death.^64^ Moreover, the higher toxicity observed following NP administration under flow conditions, compared to the platforms under static conditions highlights the key role of fluid flow in nanotoxicology investigations. In order to be able to link these observations to the administration of NPs under fluid flow, the volume of AgNP solution administered was kept constant (60 μL) in all platforms employed throughout this study, so that the dynamic administration of NPs was identified as the primary contributing factor for the observed increased toxicity. Although we have shown that immobilization of B cells (physiologically in suspension) did not induce observable toxicity response, it is likely that the antibody immobilization could alter their native state and induce activation. However, B cells anchored to glass substrate were still able to undergo cell division and maintained calcium homeostasis.^27^ Given the fact that B cells are constantly flowing in the body and that they bind to pathogens *via* specific antibodies, this CBD setup may represent a step forward towards recapitulating more physiologically-relevant shear stress.

## Conclusions

In this study, we demonstrated that the administration of AgNPs under fluid regime significantly increased their cytotoxic effect on B cells. By cross-comparing data obtained from the microfluidic device with those obtained from other commonly employed viability assays, we showed that conventional well-plate platforms under static conditions may not fully capture the adverse toxic effects to cells of the immune system. Using our microfluidic-based setup, we also investigated the effect of post-exposure time upon administration of AgNPs, which is a key parameter that should be taken into account in routine toxicity assays. In summary, to truly harness the potential of nanotechnology, we still need to better understand the interactions of NPs with biological systems and to fully characterize their potential unwanted effects. This established proof-of-concept device has the potential to become a useful tool to investigate cell–NP interactions *in vitro* and may contribute in bridging the gap between conventional *in vitro* models and animal studies.

## Author contribution

A. O. and Z. T. developed the design of the chip. A. O., M. M., T. B. S. performed experimental work. A. O. developed the data analysis methodology, wrote the manuscript. E. L., Z. T., and N. H. V. contributed to writing, review, and editing of the manuscript. Z. T. and N. H. V. developed the concept of the work and methodology and supervised the work.

## Conflicts of interest

There are no conflicts to declare.

## Supplementary Material

NA-003-D0NA00857E-s001
