# Supplementary material for: Risk assessment on-a-chip: a cell-based microfluidic device for immunotoxicity screening
Source: Nanoscale Adv. 2020 Dec 18;3(3):682–91. doi: 10.1039/d0na00857e (PMC9416880; doi:10.1039/d0na00857e)
Supplement: NA-003-D0NA00857E-s001 [file NA-003-D0NA00857E-s001.pdf]

Electronic Supplementary Material (ESI) for Nanoscale Advances.
This journal is © The Royal Society of Chemistry 2020

SUPPORTING INFORMATION

Risk assessment on-a-chip: a cell-based microfluidic device for high throughput nanotoxicity screening

Arianna Oddo[1], Mariana Morozesk[1,2], Enzo Lombi[3], Tobias Benedikt Schmidt[1,4], Ziqiu Tong[1,5,*], and Nicolas Hans Voelcker[1,5,6,7,*]

[1] *Drug Delivery, Disposition and Dynamics, Monash Institute of Pharmaceutical Sciences, Monash University, Parkville, Victoria 3052, Australia*

[2] *Universidade Federal de São Carlos, Departamento de Ciências Fisiológicas, Rod. Washington Luiz, Km 235, 13565-905 São Carlos, São Paulo, Brazil*

[3] *Future Industries Institute and UniSA STEM, University of South Australia, Mawson Lakes, 5095, South Australia, Australia*

[4] *Department of Applied Chemistry Reutlingen University, Alteburgstraße 150 72762 Reutlingen, Germany*

[5] *Commonwealth Scientific and Industrial Research Organisation (CSIRO), Clayton, Victoria 3168, Australia*

[6] *Melbourne Centre for Nanofabrication, Victorian Node of the Australian National Fabrication Facility, Clayton, Victoria 3168, Australia*

[7] *Department of Materials Science & Engineering, Monash University, Clayton, Victoria 3168, Australia*

Correspondence: Nicolas.Voelcker@monash.edu, Tommy.tong@monash.edu

Supplementary Methods

1. AgNPs quality controls via UV-Vis spectroscopy and ICP-MS

1 mL of MilliQ water was pipetted into a disposable 1.5 mL poly(methyl methacrylate) (PMMA) cuvette (Sigma-Aldrich) and a blank spectrum was collected on a spectrophotometer (Agilent 8453). 5 μL of stock AgNPs (1 mg/mL) were dispersed in the cuvette to collect the UV-Vis spectrum of the sample and to measure the localized surface plasmon resonance (LSPR). The pass/fail status of the AgNPs was determined by comparing both measured maximum absorbance and peak wavelength to reference values provided by the manufacturer.

The stability of AgNPs in cell culture media was also monitored with UV-vis. Briefly, after acquiring a blank spectrum of 1 mL complete RPMI, 5 µL of the 1 mg/mL AgNP stock solution were added to the cuvette, and UV-Vis spectra were collected every 60 min for 5 h.

2. Transmission electron microscopy and cryo-transmission electron microscopy

AgNPs from the stock solution were imaged by TEM (JEOL JEM-2100 F), equipped with a field emission gun. Sample preparation included deposition of particles on Formvar film coated copper grids (PST ProSciTech). Images were acquired at 200 kV accelerating voltage.

Cryo-transmission electron microscopy (Cryo-TEM) was used to investigate whether the size distribution of AgNPs would be affected by static incubation or administration under fluid regime. Briefly, a 1 µg/mL AgNP solution in PBS was incubated at 37°C and 5% CO_2 either in a 96-well plate, or under fluid flow in the tube connected to a syringe pump at a continuous flow rate of 0.2 µL/min for 5 h. A custom-built humidity-controlled vitrification system was used to prepare the samples for Cryo-TEM (humidity ~80% and 22°C). 200-mesh copper grids coated with perforated Lacey carbon film (ProSciTech) were glow discharged to render them hydrophilic. 3 µL of the sample were pipetted onto each grid. After 5 s, the grid was blotted for 2 s using Whatman 541 filter paper, and then plunged into liquid ethane cooled by liquid nitrogen. Samples were examined using a Gatan 626 cryoholder (Gatan, USA) and Tecnai 12 Transmission Electron Microscope (FEI, The Netherlands) at an operating voltage of 120 kV and using an electron dose of 8-10 electrons/Å^2. Images were recorded using a FEI Eagle 4kx4k CCD camera.

3. Sample preparation for ICP-MS analysis

The concentration of AgNPs and Ag^+ ions in complete RPMI medium was measured by means of ICP-MS. Microsep™ centrifuge filters (1 kDa cut-off, Pall Corporation, USA) were used to separate the AgNPs from the dissolved ions. The filters were pre-conditioned to prevent sorption of the Ag^+ ions by the membrane.[1] Briefly, 2 mL of a 0.1 M copper nitrate solution were added to the devices, which were then capped and centrifuged at 3800 x *g* for 15 min at 20°C. The membrane insert was carefully removed and excess copper nitrate solution was discarded. The device was then reassembled with a new tube, 2 mL of MilliQ water were added in the insert, which

was then capped and centrifuged as above. The rinsing water collected in the tube was discarded, and any excess water carefully removed from the insert. The procedure was repeated with 1 μg/mL AgNP solution. After centrifugation (again at 3800 x *g* for 15 min at 20°C), the dissolved ions were collected in the tube and stored at room temperature (RT) until further analysis. To attain mass balance, the AgNPs retained by the insert were etched with a solution of 20 mM $K_3Fe(CN)_6$ and $Na_2S_2O_3$ x $5H_2O$ in MilliQ water for 5 min. This treatment oxidizes Ag^0 to $Ag^+(Fe(III)(CN)_6{}^{3-})$ and the released Ag^+ ions were collected in the tube by centrifugation as previously described and stored at RT until ICP-MS analysis.

4. Detailed photolithography procedure

A chrome photomask containing transparent features (parallel rectangles, 600 μm wide and 1.3 cm long, with numbers on the top edge) was designed using the L-Edit software and fabricated using a Direct Write lithography (Intelligent micropatterning SF100). SU-8 3050 photoresist (MicroChem) was spun at 1,000 rpm on a cleaned and dehydrated 3" silicon wafer for achieving a height of 70 μm and baked at 95°C for 45 min. The wafer was then exposed to UV light through the mask (90 mJ/cm^2, EVG 6200 Mask Aligner), baked on a hot plate at 95°C for 3 min, and processed with developer to generate the layer of photoresist, which eventually formed the microchannels. SU8 developer (MicroChem) was used to dissolve away uncrosslinked photoresist.[2]

Fiji Script

Detailed script used to analyse the fluorescence images on Fiji and automatically count the number of cells at different time points. Briefly, '*Analyze Maxima*' function was employed to count the number of fluorescent cells. Other functions used for image manipulation include: '*8-bit Conversion'*, '*Smooth Function'* and '*Merge Channels'*. The example reported is for necrotic cells stained with PI:

```
//the user will select a directory containing the images of interest.
2//It will then count the cells in each image in the folder
print("\\Clear");
//Ask the user to select directory
dir = getDirectory ("Select source directory");

//Generate a list of files in selected directory
list = getFileList(dir);
Array.sort(list);

//loop the count cell code for all images in selected directory

//this loop goes through subfolders
for (i=0; i<list.length; i++){
        filename = dir + list[i];
        list_subfolders = getFileList(filename);
   Array.sort(list_subfolders);
        //for
        //Array.print(list_subfolders);
        //print(list[i]);

        //this loop goes through files
        for(j=0;j<list_subfolders.length;j++)
        {
                filename1 = filename+list_subfolders[j];
                //print(filename+list_subfolders[j]);
//check files: does it have DAPI?
                if (matches(list_subfolders[j],  ".*RFP.*")){

                        print(filename1);
                        open(filename1);
                        nameStore=getTitle();
                        rename(nameStore+" - t5h PI - "+list[i]);
                run("8-bit");
                run("Smooth");
                run("Despeckle");
                run("Find Maxima...", "noise=20 output=Count exclude");
                run("Find Maxima...", "noise=20 output=[Point Selection] exclude");
                run("Flatten");
                close();


                }
        }
}
```

Supplementary Table 1: Raw ICP-MS data used to calculate the percentage of colloidal silver and ionic silver after a 5 h incubation in cell culture medium (complete RPMI) or PBS. Limit of detection = 0.5 ppb on diluted sample = 5 ppm undiluted sample.

Sample Details					107 Ag [No Gas]	Corrected for x10 dilution	Conversion to µg/mL	Calculation of colloidal Ag in the samples		
Sample	Data File	Acq. Date-Time	Sample Name	Comment	Conc. [µg/L]	Conc. [µg/L]	Conc [µg/mL]	Average (µg/mL)	% colloidal Ag	
PBS + Ag ions 1	036SMPL.d	20/6/19 20:50	1		19.152	191.52	0.19152	0.197216667	76.73%	NP% in PBS
PBS + Ag ions 2	037SMPL.d	20/6/19 20:55	2		20.086	200.86	0.20086			
PBS + Ag ions 3	038SMPL.d	20/6/19 21:01	3		19.927	199.27	0.19927			
RPMI + Ag ions 1	024SMPL.d	20/6/19 19:46	4	<LOD	0.127	1.27	0.00127	0.0273		
RPMI + Ag ions 2	025SMPL.d	20/6/19 19:51	5	<LOD	0.206	2.06	0.00206			
RPMI + Ag ions 3	026SMPL.d	20/6/19 19:57	6		2.73	27.3	0.0273			
Ag Np etched +RPMI 1	028SMPL.d	20/6/19 20:07	7		55.74	557.4	0.5574	0.586206667	95.55%	NP% in RPMI
Ag Np etched +RPMI 2	029SMPL.d	20/6/19 20:13	8		56.328	563.28	0.56328			
Ag Np etched +RPMI 3	030SMPL.d	20/6/19 20:18	9		63.794	637.94	0.63794			
AgNP etched + PBS 1	032SMPL.d	20/6/19 20:29	10		63.149	631.49	0.63149	0.669056667		
AgNP etched + PBS 2	033SMPL.d	20/6/19 20:34	11		71.623	716.23	0.71623			
AgNP etched + PBS 3	034SMPL.d	20/6/19 20:39	12		65.945	659.45	0.65945			

Supplementary Results

Supplementary Table 2: Z-value calculation for the assays developed and employed in this study. Values are expressed as normalised viability in percentage.

	Mean of sample		SD of sample		3*SD of sample		SUM(3*SD of sample and CTRL)	|mean sample - mean CTRL|	ratio	1-ratio,
Platform	CTRL	NPs	CTRL	NPs	CTRL	NPs				Z-factor
96-well plate	100.0	5.4	0.0	3.0	0.0	8.9	8.9	94.6	0.1	0.9
PDMS wells	100.0	25.0	2.4	0.8	7.3	2.5	9.7	75.0	0.1	0.9
CBD_{static}	100.0	9.7	5.7	7.6	17.2	22.9	40.0	90.3	0.4	0.6
CBD_{flow}	100.0	20.9	0.0	5.8	0.0	17.5	17.5	79.1	0.2	0.8
$CBD_{static,P}$	100.0	26.8	0.0	17.7	0.0	53.2	53.2	73.2	0.7	0.3

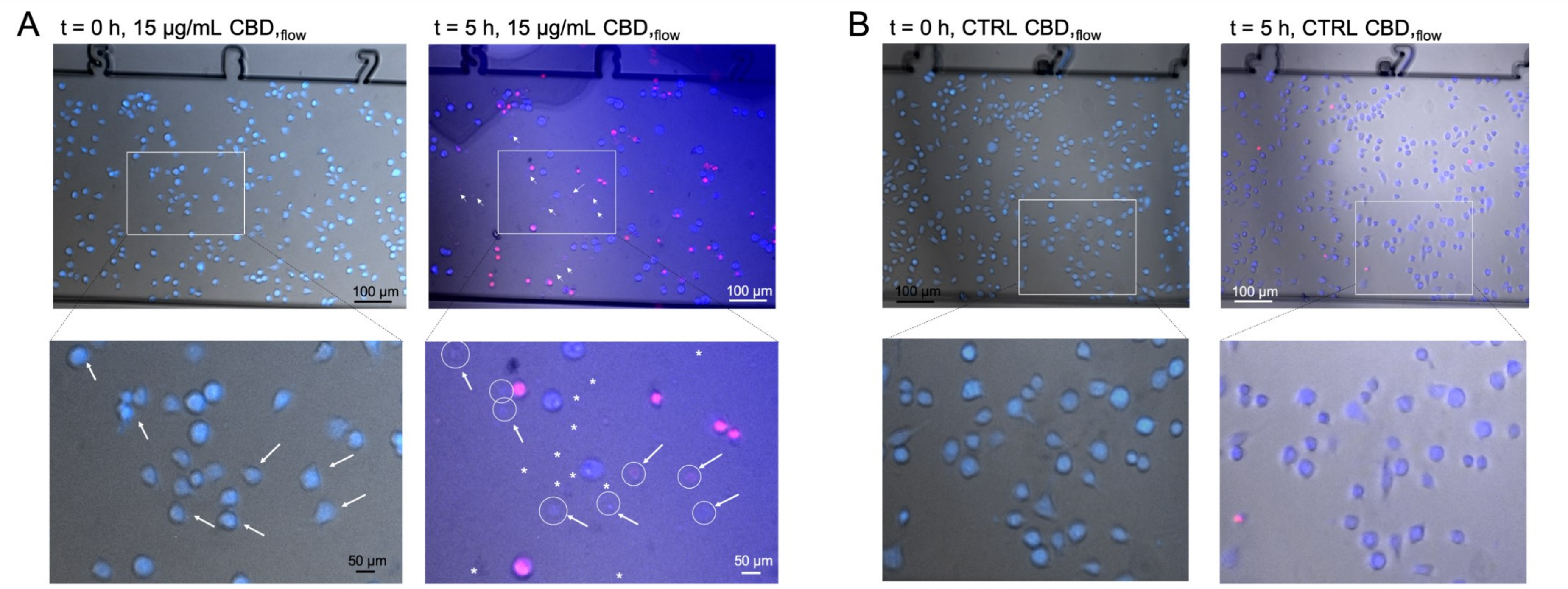

*Fig. 1: Cell detachment and membrane rupture are phenomena associated with the cytotoxicity of AgNPs. (A) The white arrows and the circles show cells that experience membrane rupture after exposure to toxic solutions of AgNPs under fluid regime (i.e., 15 µg/mL). Parts of the membranes and of the nuclei can be seen inside the white circles, as a faded blue/red staining. Cells that detached after exposure to AgNPs are indicated with *. Both cell with a compromised membrane and cells that detached are counted as missing cells in this study. (B) the exposure of HR1K cells to cell culture media without AgNPs did not cause any cells to detach or membrane rupture.*

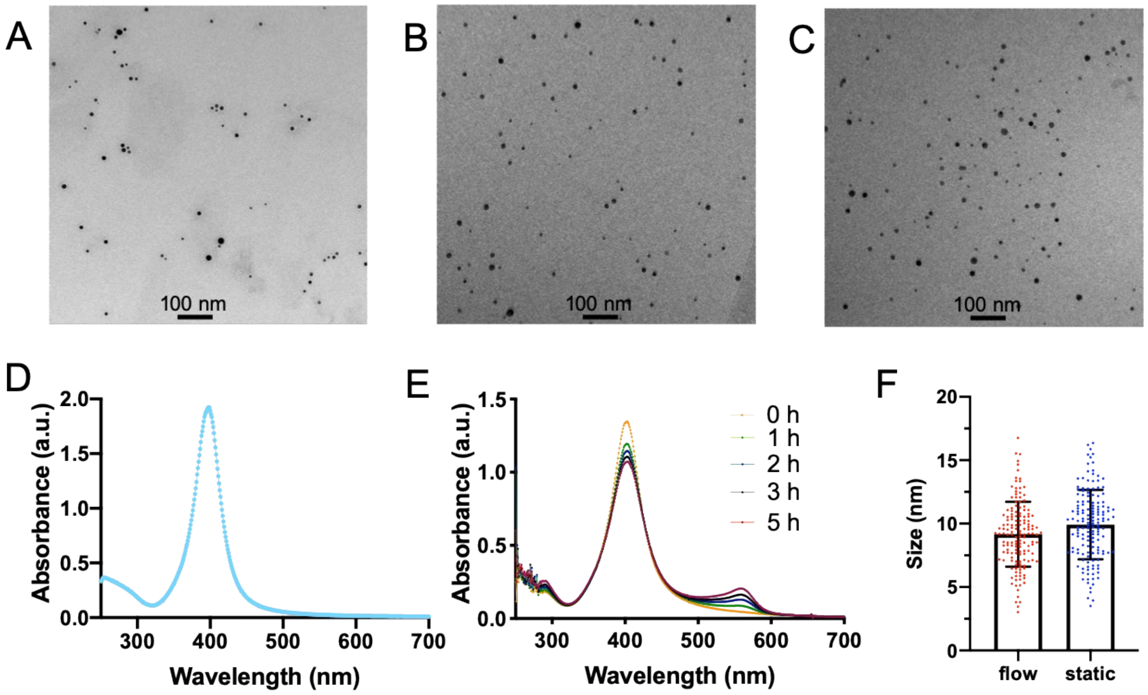

Fig. 2: Characterization of the stock AgNPs solution and stability. (C) TEM images of 10 nm AgNPs in ultrapure water, (B) Cryo-TEM images of AgNPs after incubation in PBS for 5 h under static conditions and (C) under flow conditions. (D) UV-Vis spectra of AgNPs in MilliQ water, (E) UV-Vis spectra of AgNPs in complete medium, acquired every hour over a period of 5 h, and (F) measured diameter for 150 AgNPs measured from CryoTEM images upon static and flow conditions.

References

1. P. Wang, E. Lombi, S. Sun, K. G. Scheckel, A. Malysheva, B. A. McKenna, N. W. Menzies, F.-J. Zhao and P. M. Kopittke, *Environmental Science: Nano*, 2017, **4**, 448-460.
2. Z. Tong, A. Ivask, K. Guo, S. McCormick, E. Lombi, C. Priest and N. H. Voelcker, *Lab on a Chip*, 2017, **17**, 501-510.
